# The Impact of Out-of-Pocket Payments on Health Care Inequity: The Case of National Health Insurance in South Korea

**DOI:** 10.3390/ijerph110707304

**Published:** 2014-07-18

**Authors:** Weon-Young Lee, Ian Shaw

**Affiliations:** 1 Department of Preventive Medicine, College of Medicine, Chung-Ang University, 84 Heukseok-Ro, Dongjak-Gu, Seoul 156-756, Korea; 2 School of Sociology and Social Policy, University of Nottingham, University Park, Nottingham NG7 2RD, UK; E-Mail: Ian.Shaw@nottingham.ac.uk

**Keywords:** health care utilization, equity, catastrophic health expenditure, social health insurance, out-of-pocket payments

## Abstract

The global financial crisis of 2008 has led to the reinforcement of patient cost sharing in health care policy. This study aimed to explore the impact of direct out-of pocket payments (OOPs) on health care utilization and the resulting financial burden across income groups under the South Korean National Health Insurance (NHI) program with universal population coverage. We used the fourth Korean National Health and Nutrition Examination Survey (KNHNES-IV) and the Korean Household Income and Expenditure Survey (KHIES) of 2007, 2008 and 2009. The Horizontal Inequity Index (HIwv) and the average unit OOPs were used to measure income-related inequity in the quantitative and qualitative aspects of health care utilization, respectively. For financial burden, the incidence rates of catastrophic health expenditure (CHE) were compared across income groups. For outpatient and hospital visits, there was neither pro-poor or pro-rich inequality. The average unit OOPs of the poorest quintile was approximately 75% and 60% of each counterpart in the richest quintile in the outpatient and inpatient services. For the CHE threshold of 40%, the incidence rates were 5.7%, 1.67%, 0.72%, 0.33% and 0.27% in quintiles I (the poorest quintile), II, III, IV and V, respectively. Substantial OOPs under the NHI are disadvantageous, particularly for the lowest income group in terms of health care quality and financial burden.

## 1. Introduction

Historically, the expansion towards “universal coverage for health care” suggested by the World Health Organization has occurred in three main ways. First, the proportion of the population that enjoys social health protection increases; second, an expansion of the range of essential services occurs; and/or third, a reduction of cost sharing. Each factor has been regarded as a desirable goal for health care systems [1,2,3,4]. High income countries have achieved this goal in a range of different ways and many middle or low income countries have also made efforts to attain to this goal [4,5]. However, the use of direct user payments in healthcare remains controversial, in regard to whether it should exist at all, and if it does exist, at what level it should be charged [6]. According to the 2010 World Health Report [4], because millions of individuals cannot utilize health services or suffer financial hardship because of direct payments at the time health care service are received; moving away from direct payments is an important step to avert the financial hardship associated with paying for health care. Moreover, Jeffrey Sachs’ United Nations report called the abolition of user fees a “quick win” in speeding up progress towards the health Millennium Development Goals [7]. On the other hand, it continues to be advocated that a certain amount of direct user payments is useful to align consumers’ incentive in situations of moral hazard so that over-utilization of medical services is mitigated [8,9]. This debate has come to the fore in the wake of the economic recession following the financial crisis of 2008 [10].

South Korean National Health Insurance (NHI) has often been praised for its rapid achievement of universal population coverage compared with several high income countries [11]. However, the proportion of direct out-of-pocket payments (OOPs) of the total health care cost has remained between 32%–36%; in the previous decade (2001–2011), these costs were substantially higher compared with the Organization for Economic Co-operation and Development (OECD) average of approximately 20% [12] despite the extension of coverage of the South Korean NHI to all individuals in 1989. Thus, South Korea represents a good country to assess the adverse equity effects of the patient cost sharing arrangement under public health insurance. Experiences obtained from South Korea can provide valuable information to countries where a greater level of cost-sharing for health care is considered as an alternative to strengthen the sustainability of tax-funded or public health insurance systems. 

Table 1 shows the institutional characteristics of the South Korea NHI. The South Korean NHI funds primarily originate from individual member contributions, which amount to approximately 80% of the fund, and the central governmental budget is responsible for the remaining fund [13]. The proportion of cost sharing for comprehensive health care (e.g., inpatient and outpatient services, prescription drugs and dental care) was approximately 35% between 2007–2011 [14] which included 13% of copayments for covered services by the NHI and 22% of the full cost for non-covered services. Health care providers are allowed extra-billing for the provision of these uninsured services which are bundled with insured services during the same hospital care episode or clinic visit. These non-covered services include new and high-cost technologies despite medically necessary care and surcharge rates to basic fees at large hospitals. To prevent the negative effect of this substantial cost sharing on individuals with vulnerable conditions’ (e.g., some catastrophic diseases, orphan diseases, aged 6 or less), direct copayments for covered services have been substantially lower compared with other individuals and the ceilings of copayments for insured services have been employed with consideration for a patient’s income level, as shown in Table 1. However, these arrangements have not been applicable to services that are not covered by the NHI. For example, the total annual cost of hospital treatment for a cancer patient is $10,000 ($7000 for covered service and $3000 for non-covered service), the direct use payments for covered service is $350 (copayment rate: 5% for covered service), and the direct payments for non-covered services is $3000 (when paid in full). In this case, the ceiling of copayments is not beneficial despite direct OOPs that amount to $3350 in reality. Consequently, those protection arrangements against catastrophic expenditure are not effective in relieving the patient’s financial burden as much as expected. In addition, the Medicaid-Aid program (MAP) for individuals below the poverty line (approximately 3% of the entire population) has provided covered service by the NHI at a substantially lower expense compared with the insured pay. However, health care services outside of the NHI have also placed their full cost on patients despite MAP beneficiaries. Approximately 90% of health care facilities in South Korea are privately owned, and the health care service delivery system is very competitive without state imposed competitive regulation. Moreover, most health care services and drugs are reimbursed by fee-for-service arrangements. These characteristics could represent the reason for the encouragement of health care providers to increase their service intensity and/or the use of non-covered medical services such as high-cost health technology.

This study, first set out to identify the influence of direct OOPs on health care utilization within and between income groups. Equity in health care utilization was evaluated in both outpatient and hospital visits, which indicated contacts to health care system and differences in the amount of OOPs as a proxy for used service volume and the consumption of non-covered services such as high-cost health technology service. Second, we evaluated how the direct OOPs have affected socio-economic groups using information regarding the incidence of households with catastrophic health expenditures and impoverishment that resulted from paying for health care. 

**Table 1:** Institutional structure of South Korean National Health Insurance.

Institutional Structure	Contents
Financing resources and organization	Compulsory social health insurance based contribution (80%)
	Government budget subsides (20%)
	Single fund/insurer (South Korean National Health Insurance Corporation, NHIC)
Population Coverage	About 97% of total population
	The remaining population (about 3%) below the poverty line supported by Medical-Aid Program (MAP)
Benefit coverage	Most curative services and drugs
	Some non-covered services despite being medically necessary: new services, high-technology care, surcharges to regulated fees at large hospitals
Out-of-pocket payments (e.g., As of 2009, direct OOPs accounted for about 35% of total treatment cost at the use of health care service which comprised 22% as the full payment for of non-covered service and 13% as copayments for covered service)	Covered services: copayment rates and total cost - Inpatient service: 10%–20% - Outpatient service: 30% (clinics, pharmacy), 40% (hospitals), 50% (general hospitals), or 60% (tertiary care hospital) Non-covered services - Paid in full at market-based price - No case exceptions (e.g., beneficiaries for MAP and patients with catastrophic conditions must pay full cost for medical service received) - Surcharge rates to basic fees at large hospitals - New and high-cost technology (drug) - Cosmetic service Some protection measures from high OOPs - 5% copayment rates for covered services designed to treat several catastrophic conditions: cancer, or severe cardiovascular events - No or discounted copayments for covered services for MAP beneficiaries - Ceilings on the cumulative copayments that exclude the cost for non-covered service incurred within more than 6 consecutive months for covered services: the lower half of households ($1818), the next 30% ($2727), and the top 20% ($3636)
Health care provider payment mechanism	Fee for service payments (in principal) Prospective payments based on the Diagnostic Related Group (DRG) for several diseases, voluntary participation of hospitals
Health care delivery systems	About 90% of health care services provided by private sector No gatekeeper system for the coordination among providers Patients’ unconstrained freedom in choose providers & competitive settings

## 2. Methods

### 2.1. Data Source

We used data from the fourth Korean National Health and Nutrition Examination Survey (KNHNES-IV) of Korea’s Center for Disease Control and Prevention (Table 2). This survey was a continuous program with new sample of about 10,000 individuals each year except for the year 2007, when the number of participants was half of that of other years (2008 and 2009) as the 2007 survey was conducted during a half-year (from July through December). The KNHNES-IV comprised representative information regarding the Korean population related to health care service use and OOPs for the use of health care services. For health care utilization, the survey contained self-reported information regarding the frequency of visits to physician’s offices or outpatient clinics at hospitals in the previous two weeks and the frequency of admission to an acute hospital in the previous year. These window periods were chosen considering the recall bias of self-reported survey and KNHNES-IV has been conducted all the year round without a designated survey duration.

These data were used to analyze income-related inequalities in health care utilization and gaps in OOPs between income groups. Although the fifth KNHNES was conducted in 2010, 2011 and 2012, the data could not be used for this study, because they did not contain information regarding direct OOPs for the use of health care services. However, there was no noticeable change in cost sharing policies between 4th and 5th survey years of the KNHNES. In addition, dental care was excluded from the current analysis, because this study focused on medical care. The Korean Household Income and Expenditure Survey (KHIES) of 2007, 2008 and 2009 were used to analyze the incidence rates of households with catastrophic health expenditures in different income groups and to identify the impoverishment that resulted from co-payments. 

### 2.2. Measurement & Statistics

#### 2.2.1. Differences in Health Care Utilization among Income Groups

To measure income-related horizontal (in)equality (HI) in outpatient visits and hospital admissions, we employed HIwv suggested by Wagstaff and van Doorsler [15,16]. The HIwv index assesses the extent to which South Korean individuals with low income have the same access to necessary health care services compared with more affluent groups and is fitted with micro data [15,16]. This factor is derived from a concentration (C) index in the use of health care, after standardizing the need for health care [15,16]. The C index is the measure of relative income-related inequality in health care use and lies in the range of (−1–1), with a positive (negative) sign indicating pro-rich (pro-poor) inequality (see Appendix). Using the indirect standardization approach to adjust the need of health care to the C index which measures the relative income-related inequality in health care distribution, the Horizontal Inequity (HIwv) can be generated, and it lies in the range of (−2–2), with a positive (negative) value indicating pro-rich (pro-poor) inequity (see Appendix). This study used ordinary least square regression (convenient regression) to estimate the stand error of C and HIwv indexes with micro data (see Appendix). 

#### 2.2.2. Differences in OOPs per Clinic Visit and per Inpatient Day among Income Groups

According to van Doorslaer and Masseria [15], the HIwv based on the frequency of outpatient visits and hospital admissions is unable to clarify the exact nature of a visit or an admission or the intensities of care because the HIwv adopts a default assumption that a visit is a visit. We hypothesized that the amount of direct OOPs per outpatient visit and admission episode to hospital indicate the quality of health care in the health care policy context of South Korea. This is because the NHI has maintained relatively high copayment rates for covered services and a wide range of health care services and new drugs outside of the NHI benefit package regardless of their medical necessity. If there is a significant difference in direct OOPs that pay for health care services between high and low income groups, it could indicate that the income group that pays relatively less OOPs does not receive sufficient health care to recover from diseases and other medical conditions. 

It would be biased to make a simple comparison of the average OOPs per outpatient visit and per admission day among the five income groups without controlling for influential variables on health care cost. We used an analysis of covariance (ANCOVA) model to examine the adjusted differences between the health care cost means of five independent groups by equivalent household income under the control of confounding variables, such as sex, age, Medicaid-Aid recipient status, residential area (urban *vs*. rural), private health insurance, and self-rated health status. To fulfill the assumption of the ANCOVA model that the dependent variable (direct OOPs) of each income group is normally distributed, a logarithmic transformation of these average costs was conducted to ensure a closer to normal distribution. For the ANCOVA, we ran univariate General Linear Model (GLM) in IBM-SPSS 21.0. Households were divided into five income groups of equal size (20%) based on the equivalent household income using the old OECD equivalence scales of 1:0.7:0.5.

#### 2.2.3. Catastrophic Health Care Expenditure and Impoverishment that Results from Paying for Health Care

Several indices to measure the CHE have been proposed by different researchers in different settings. According to Wagstaff and van Doorsaler’ s suggestion [17], we used thresholds of 10%, 20%, 30% and 40% of the capacity to pay, which was defined as the income that remained after subsistence needs had been met. In practice, this value amounts to the total household expenditure minus the food expenditure. Various thresholds, including a conventional threshold of 40%, were used to conduct an informative comparison of the financial burden on the family budget between socio-economic groups. Another approach for measuring the financial burden of OOPs on households was to determine how many households were impoverished by the direct OOPs on health care. The South Korean national minimum living cost based on the number of family members annually, which was announced by the Korean Health and Welfare Ministry during the study period, was used as a poverty line. If a household’s pre-payment living costs or post-payment living costs after paying OOPs was below this minimum, the household was classified as in poverty. Differences between the incidence rates of post-payment poverty and pre-payment poverty indicated the impoverishment that resulted from direct OOPs. Households were therefore divided into five income groups.

## 3. Results

### 3.1. Differences in Health Care Service Use among Income Groups

Table 3 presents the C and HIwv indices for the average outpatient clinic and hospital visits during 2007–2009. The average number of outpatient visits was 0.549. The C index based on actual use (*C_m_*) in outpatient visits, which was not adjusted for differences in health care need or other factors in income groups was −0.114 (95% CI, −0.134–−0.094) in 2007, which indicates significant pro-poor inequality. After adjusting for various influential factors including health care need, the negative correlation between outpatient visits and income group disappeared, which was indicated by the HIwv index of was −0.006 (95% CI, −0.025–0.014) during 2007–2009. This result implies that there was no inequality in outpatient visits between those income groups. 

**Table 2:** Details of data resources.

Survey	Year	Sample Size	Response Rate (%)	Inclusion Criteria	Survey Content
Fourth National Health and Nutrition Examination Survey (KNHNES-IV)	2007	4594 (individual)	71.2	Individuals aged 20 and over except for dental clinic or hospital admission	Demographic characteristics Socioeconomic characteristics Self-rated health status
					Frequency of visit to physician’s offices, clinics or outpatient clinics at hospitals in the previous two weeks (self-reported)
	2008	9744 (individual)	77.8		Frequency of admission to an acute hospital in the previous year (self-reported)
	2009	10,533 (individual)	82.8		Direct out-of-pocket health care payments for the use of outpatient and inpatient services
Korean Household Income and Expenditure Survey (KHIES)	2007	8700 (households)	80	Households in urban areas and with family numbers of two or more	Household status with the number of family members, occupation and industry of the head of household and the spouse, the type of dwelling, *etc*.
	2008	8700 (households)	80		
	2009	8700 (households)	80		Household income related items Household expenditure related items including food and medical service

The average number of hospital admissions during the previous year was 0.113 over 2007, 2008 and 2009. The C for actual hospital admissions showed pro-poor inequality at −0.073 (95% CI, −0.104–−0.042) in 2007, however after adjusting for health care need, the negative correlation (pro-poor equality) between hospital admissions and income groups disappeared, and the HIwv index was 0.007 (95% CI, −0.024–0.037). The HIwv in hospital admissions did not show a significant negative (pro-poor inequality) or positive relationship (pro-rich inequality) even though the C Index in actual use had pro-poor inequality (see Table 3). 

**Table 3:** Concentration and Horizontal Inequity Indexes in the average outpatient visits and the average number of hospital admissions during 2007–2009.

Service Category	Outpatient			Inpatient		
Average Frequency of Clinic Visits or Hospital Admissions)	0.549			0.113		
Parameters	Estimates	95% CI		Estimates	95% CI	
*C_m_* (actual use)	−0.114	−0.134	−0.094	−0.073	−0.104	−0.042
*C_n_* (need-expected use)	−0.106	−0.111	−0.102	−0.079	−0.083	−0.076
HIwv ( use after standardizing the differences in need and others *)	−0.006	−0.025	0.014	0.007	−0.024	0.037

Note: * Need and other factors: age, sex, residential area (urban *vs*. rural), Medicaid Aid recipient, private health insurance and self-rated health status.

### 3.2. Differences in OOPs per Outpatient Visit and per Inpatient Day among Income Groups

Table 4 below shows the average OOPs per outpatient visit and hospital admission day in all income groups as well as the ratios of each group (I, II, III, IV) to the richest group (V) during 2007–2009, using analysis of covariance (ANCOVA). The average unit OOPs were significantly lower in the second (II) and poorest (I) quintiles compared with the richest quintile (V). For example, the ratios of the average OOPs per clinic visit of the poorest (II) and second (I) quintiles compared with the richest quintile (V) were 0.83 (95% CI 0.74–0.94) and 0.75 (0.66–0.85), respectively. The average OOPs per inpatient day were significantly lower only in the poorest quintile (I) compared with the richest quintile (V). The ratio of the hospital average costs per day of the poorest quintile (I) compared with the richest quintile (V) was 0.59 (95% CI 0.51–0.68).

**Table 4:** The average unit OOPs (per outpatient visit and inpatient day) and the ratios of each group (I, II, III, IV) to the richest group (V) during 2007–2009.

Service Category	Outpatient					
Income Group	No.	Mean	SD	Median	Ratio	95% CI
V	728	7.74	8.94	3.96	1.00	
IV	679	6.90	8.17	3.66	0.89	0.79–1.00
III	755	6.43	7.25	3.76	0.93	0.82–1.05
II	803	5.63	6.80	3.41	**0.83**	**0.74–0.94**
I	990	4.73	6.57	2.03	**0.75**	**0.66–0.85**
Total	3955	6.16	7.58	3.49		
**Service category**	**Inpatient**					
**Income Group**	**No.**	**Mean**	**SD**	**Median**	**Ratio**	**95% CI**
V	270	193.52	317.55	126.67	1.00	
IV	279	178.78	255.15	123.33	1.00	0.85–1.19
III	243	158.12	186.51	101.16	0.87	0.73–1.04
II	251	147.75	188.82	97.43	0.91	0.76–1.08
I	294	181.57	460.79	76.67	**0.59**	**0.51–0.68**
Total	1337	172.79	306.53	101.25		

Notes: Mean, SD, Median: thousand won, 1 U$ = 1100 thousand won; Ratio: ratios of the geometric mean of each group (II,III,IV,V) compared with the poorest group (I), adjusted for sex, age, Medicaid Aid recipient, private health insurance, residential area and self-reported health status, using Analysis of covariance (ANCOVA) with logarithmic transformation of the average unit (per clinical visit, per inpatient day) cost.

### 3.3. Distribution of Households with Catastrophic Health Expenditure across Income Groups

**Figure 1:**
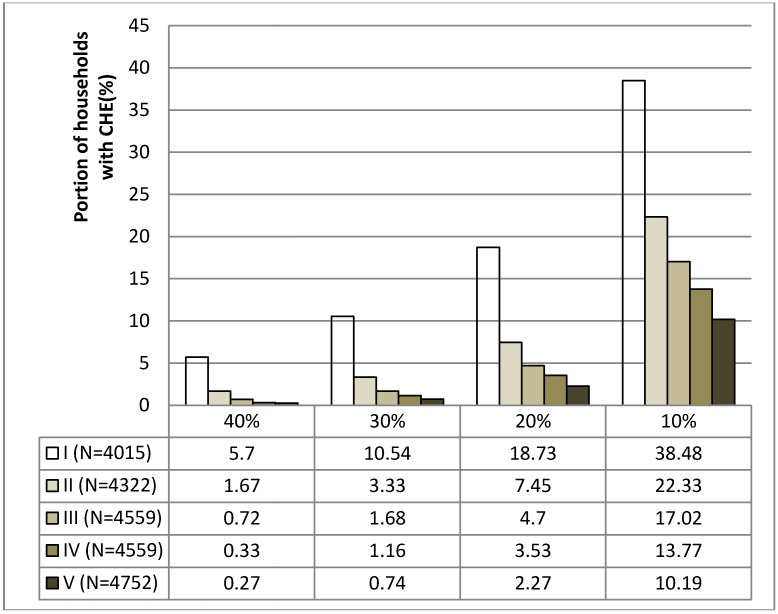
The average income related distribution of households with catastrophic health expenditure by the thresholds of 10%, 20%, 30% and 40% during 2007–2009.

**Figure 2:**
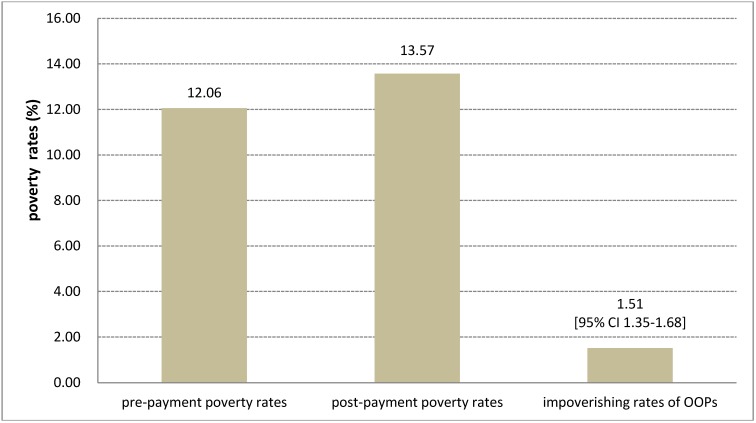
The average **i**mpoverishment rates of OOPs during 2007–2009.

Figure 1 shows the income-related distribution of the CHE incidence rates by the various thresholds in 2007. The lower the household income, the higher the incidence of households with CHE after paying OOPs for health care in all thresholds (10%, 20%, 30% and 40%). For example, during 2007–2009, when the threshold of CHE was 40%, the average incidence rates were 5.7%, 1.67%, 0.72%, 0.33% and 0.27% in quintiles I (the poorest quintile), II, III, IV and V (the richest quintile), respectively. As shown in Figure 2, the average impoverishing rate of OOPs after paying for health care was 1.51% (95% CI 1.35–1.68) during the same period, which implies that paying for health care expense made approximately 1.5% of the total households fall below the poverty line. 

## 4. Discussion and Conclusions

Low-income individuals did not appear to have more difficulty in establishing contact with a physician or hospital compared with individuals with higher incomes. However, the implications from the findings are that the poorest quintile group was less likely to be provided with sufficient or advanced health care services compared with the richest quintile group. Moreover, the CHE incidence rate in the poorest group was approximately 20 times higher compared with in the richest group.

A significant HI was not observed in outpatient visits or admissions to the hospital. Given that the patient cost sharing rates were approximately 35% of the total expense of health care which included both covered and non-covered services, these results were inconsistent with the fact that high OOPs and a certain number of uninsured individuals regressively led income-related health care utilization in a pro-rich direction indicated by a positive HIwv as shown in the United States [18] and Mexico [19]. 

There are several possible explanations for our results. For overall outpatients’ visits, a study by Lu *et al*. [20] that computed the HIwv using the data from the first KNHNES in 1998 reported the same results as our study. The study calculated the HIwv of outpatient visits according to the type of health care facilities. As a result, public health centers showed a significant pro-poor tendency, while pro-rich inequity was present in the use of outpatient clinics at tertiary-level medical institution in South Korea. This finding is because South Korean public health centers have traditionally provided outpatient services to vulnerable individuals in urban areas and to all rural residents at substantially lower copayment rates than required by outpatient clinics at large hospitals. In this study, we could not compute the HIwv of clinic visits by each type of health care facility because the relevant data in the KNHNES-IV was collected without the distinction of the type of health care facility, which was different compared with KNHNES-I. Further analyses of the HIwv of clinic visits according to the type of health care facility (e.g., physician office, outpatient clinic at a general hospital or tertiary hospital) or health care service (e.g., medical care or dental care) are necessary when data is available. 

For inpatient services, our results did not show income-related horizontal inequality in inpatient service use. In addition, Lu *et al*. [20] presented pro-poor inequality in the use of inpatient services by the HIwv index in South Korea. This finding implies that despite substantial direct OOPs for inpatient services, pro-rich inequity has not been observed, at least in terms of the quantitative aspects of hospital admissions. This result may be attributed, in part, to the universal population coverage of the South Korean health security system. In particular, the NHI has assured the right of access to health care for the enrolled if they intend to pay social health insurance contributions. On the other hand, the United States and Mexico have a larger number of uninsured individuals [18,19,21,22]. These differences might lead to different effects on income-related health care use in terms of contact with the health care system. 

Average unit OOPs that represented service intensity and consumption levels of non-covered services, such as high-cost technology services, were significantly lower only in the poorest quintile compared with the richest quintile. South Korean cancer patients with low incomes have been treated at a lower service intensity or volume and have used tertiary-level hospitals less frequently compared with individuals with high incomes [23,24]. This trend implies that patients in the poorest quintile might not receive adequate quality health care services to recover from illness.

The disproportionate concentration of the CHE incidence in the poorest population quintile was observed in 2007. This observation may be due to the substantial patient cost-sharing arrangement, which has insufficient consideration of a patient’s ability to pay, and which has been embedded in the South Korean NHI for a long time. For example, the ceiling scheme that does consider a patient’s income level is applicable only to services covered by the NHI and not to non-covered services. For these non-covered services, the health care providers have been allowed to charge patients at full cost of them regardless of the patients’ income, thereby resulting in the worse-off (including beneficiaries for MAP) having to use non-covered services for treatment. Consequently, disparities in the concentration of the CHE incidence among income groups were perhaps not surprising in the context of the structure of the South Korean NHI. 

This study has several limitations of the assessment of inequity in health care utilization and the financial burden of the South Korean health security system. First, an analysis of income-related health care utilization by individual types of health care facilities would have been useful because the levels of cost-sharing rates are different among facilities. However, the KNHNES-IV did not contain the relevant records according to types of health care facilities (e.g., physician office, hospital, public center). Second, in general, health-related indicators, such as mortality rates and biomarkers have been used as indicators of health care quality. Instead, this study used gaps in direct OOPs by income group because of a lack of relevant national data. 

Some studies [25,26] have reported that more direct OOPs would not assure a higher quality of health care in the United States. However, gaps in OOPs in this study could reflect, in part, difference in quality of health care in the South Korean policy context [27,28]. Finally, the catastrophe incidence that results from high direct payments by income group may be more pronounced in patients with chronic diseases or catastrophic diseases. However, the KHIES did not contain sufficient health-related information for its participants. 

Since this study did not examine rigorously a causal relationship between high OOPs and inequity in health care, our findings should be interpreted very carefully. However, these experiences of the South Korean NHI could leave some implications to countries struggling on the path to universal health care. First, since the South Korean NHI has universal population coverage, there might be seen no income-related horizontal inequity in clinic visits and hospital admission despite of high out-of-pocket payments. However, this study demonstrates that high out-of-pocket payments could impose low quality health service and catastrophic financial burden on the lowest quintile group among all enrollees of the South Korean NHI. Consequently, the expansion for health care coverage should be made toward service and cost as well as population to achieve universal health coverage [4,29,30,31]. Second, although twenty years have passed since full population coverage began, the South Korean NHI still has not reached the full concept of universal coverage in terms of service and cost coverage. These results demonstrate a potential pitfall of the “population coverage route”, *i*.*e*., to have all individuals enrolled in the NHI prior to service [27]. Given that different strategies have different shortcomings, the choice of the most efficient strategy to achieve universal coverage should be made based on each country’s individual context. 

## Appendix

Let *y_i_* denote the amount of health care received by individual *i* in a given period. The inequality in the distribution of medical care by income is captured by the medical care concentration curve *L_M_ (R)* which graphs the cumulative proportion against the cumulative proportion R of the sample ranked by income. The *C_m_* index, corresponding to *L_M_ (R)* indicates the degree of inequality in the distribution of medical care and can be measured as twice the area between *L_M_ (R)* and the diagonal, or equivalently as [12]:

(1)

However, because *C_m_* does not consider the differences in health care need among income groups it cannot reflect an income related horizontal inequality (HI) in health care utilization. According to the method of the indirect standardization of health care need we can generate a predicted value of health care use for each individual *i* which indicates the amount of health care the individual would have received if she had been treated as others with the same need characteristics (e.g., sex, age, self-reported health state). This value can be considered an individual’s health care need and the *C_n_* index of health care need based on the concentration curve of need, labeled *L_N_ (R)*, as follows:

(2)

The proposed measure of *HIwv* is defined as twice the area between the need and actual health care concentration curves and can simply be computed as the differences between *C_m_* and *C_n_* [16]:

(3)

A positive (negative) value of *HIwv* indicates horizontal inequity that favors the better-off (worse-off). A zero index value indicates no HI. *L_M_ (R)* is a function from 0 to 1, which is monotonically increasing. Consequently, the area under *L_M_ (R)* is between 0 and 1 such that *C_m_* is between −1 and 1. The same applies to *C_n_*, and then *HIwv* can be between −2 and 2.

Estimate for the C index in the actual use in health care (*C_m_*) and its robust standard error are obtained by running the following ordinary least square (OLS) regression [15,32]:

(4)

where *y_i_* is health care use (outpatient and inpatient services), *y^m^* is its mean , *β* is the estimate of *C_m_*, *R_i_* is the weighted relative fractional rank of the *i*th individual in the income distribution , where w_i_ is the sampling weight, w_0_ = 0 and N is the sample size) and  is the weighted variance of *R_i_*_._. The rank of an individual out of the sample in the income distribution was determined by the equivalized income of his or her household using old OECD equivalence. This approach was used to assigns a value of 1 to the first household adult member, a value of 0.7 to each additional adult and a value of 0.5 to each child aged 14 or less.

To account for differences in the need for health care the following “demand” function for health care utilization was estimated [15,32]:

(5)

where: *y*_i_ is the number of outpatient and hospital visits during the study period by individual *i*; *ln*(*inc_i_*) is the log of the individual *i*’s household equivalized income; *X_k_* are variables that represent the need for health care (age, gender, self-rated health status); *Z_p_* are other variables that influence demand for health care (residential area, Medicaid Aid recipient, private health insurance); and *α*, *β*, *γ_k_*, *δ_p_* are parameters. The expected number of outpatient and hospital visits was then identified for an individual *i* at the values of need variables *X* given other characteristics *z* at their sample mean values [11,27]:

(6)

The need-standardized utilization rates were derived as follows [15,32]:

(7)

where *y^m^* is the average frequency of outpatient and hospital visits in the sample. The *HIwv* and its standard errors were calculated similarly from the following model:

(8)

where *β* is the estimate of the need-standardized concentration index or *HIwv* index.
